# Human Herpesvirus-6 Induces MVB Formation, and Virus Egress Occurs by an Exosomal Release Pathway

**DOI:** 10.1111/j.1600-0854.2008.00796.x

**Published:** 2008-08-26

**Authors:** Yasuko Mori, Masato Koike, Eiko Moriishi, Akiko Kawabata, Huamin Tang, Hiroko Oyaizu, Yasuo Uchiyama, Koichi Yamanishi

**Affiliations:** 1 Laboratory of Virology and Vaccinology, Department of Biomedical Research, National Institute of Biomedical Innovation7-6-8, Saito-Asagi, Ibaraki, Osaka 567-0085, Japan; 2 Division of Clinical Virology, Kobe University Graduate School of Medicine7-5-1, Kusunoki-cho, Chuo-ku, Kobe, 650-0017, Japan; 3 Department of Cell Biology and Neurosciences, Osaka University Graduate School of Medicine2-2 Yamadaoka, Suita, Osaka 565-0871, Japan; 4 Department of Cell Biology and Neurosciences, Juntendo University School of Medicine2-1-1 Hongo, Bunkyo-ku, Tokyo 113-8421, Japan

**Keywords:** budding and egress, exosome, final envelopment, HHV-6, MVB, TGN

## Abstract

The final envelopment of most herpesviruses occurs at Golgi or post-Golgi compartments, such as the *trans* Golgi network (TGN); however, the final envelopment site of human herpesvirus 6 (HHV-6) is uncertain. In this study, we found novel pathways for HHV-6 assembly and release from T cells that differed, in part, from those of alphaherpesviruses. Electron microscopy showed that late in infection, HHV-6-infected cells were larger than uninfected cells and contained many newly formed multivesicular body (MVB)-like compartments that included small vesicles. These MVBs surrounded the Golgi apparatus. Mature virions were found in the MVBs and MVB fusion with plasma membrane, and the release of mature virions together with small vesicles was observed at the cell surface. Immunoelectron microscopy demonstrated that the MVBs contained CD63, an MVB/late endosome marker, and HHV-6 envelope glycoproteins. The viral glycoproteins also localized to internal vesicles in the MVBs and to secreted vesicles (exosomes). Furthermore, we found virus budding at TGN-associated membranes, which expressed CD63, adaptor protein (AP-1) and TGN46, and CD63 incorporation into virions. Our findings suggest that mature HHV-6 virions are released together with internal vesicles through MVBs by the cellular exosomal pathway. This scenario has significant implications for understanding HHV-6's maturation pathway.

Human herpesvirus 6 (HHV-6) belongs to the betaherpesvirus genus *Roseolavirus*. HHV-6 was first isolated from the peripheral blood lymphocytes of patients with lymphoproliferative disorders and acquired immune-deficiency syndrome (1). HHV-6 isolates are categorized as two variants, A (HHV-6A) and B (HHV-6B), on the basis of their *in vitro* growth properties, DNA restriction site polymorphisms, antigenicity and host-cell tropism (2–5). HHV-6B is the causative agent of exanthem subitum (6), but the role of HHV-6A in human disease is less clear.

The herpesvirus family has many members, which share virion architecture, general aspects of the replicative cycle and a small number of conserved genes (7). They differ in their sites of latency, the function of many genes and the specific details of their replicative cycle. It is now established that viral capsids mature by acquiring a layer of proteins designated as the tegument and a membrane structure containing virus-specific glycoproteins (7). This glycoprotein-containing envelope is found both at the inner nuclear membrane and in the cytoplasm. Mature herpesvirus particles are thought to be transported to the extracellular space by the fusion of a virus-containing vesicle with the plasma membrane (8). For some alphaherpesviruses, the final envelopment in the cytoplasm occurs at the membrane of the *trans* Golgi network (TGN) or TGN-derived vesicles (9–15). It has been suggested that human cytomegalovirus (HCMV), which belongs to the betaherpesviruses, is also enveloped at this site (16,17). Other studies have proposed a role for the endocytic membrane in HCMV envelopment (18–20). In addition, large accumulations of viral components in the perinuclear region function as cytoplasmic virus factories (21). However, the precise organization and dynamics of such cytoplasmic factories are not completely understood.

Among the herpesviruses, the mechanisms of HHV-6 virion maturation and egress are particularly poorly characterized. A recent electron microscopic study demonstrated that HHV-6 nucleocapsids in the cytoplasm acquire a thick tegument and a final envelope containing viral glycoproteins at the level of newly formed annulate lamellae (AL) or at the *cis* side of the Golgi complex (22), which is a different site from the other herpesviruses. More recently, another group showed AL in the cytoplasm of HHV-6A-infected cells, but at low levels, with the final envelopment of HHV-6 viral particles occurring in cytoplasmic vesicles (23). Therefore, in this study, we performed experiments to identify and characterize the precise routes of intracellular virus maturation and egress associated with HHV-6 infection.

In this study, we found that the cytoplasmic envelopment of HHV-6 takes place in TGN- or post-TGN-derived membranes that contain, in addition to viral glycoproteins, the cellular proteins CD63 and clathrin. During the course of infection, enveloped virus particles were observed in membrane structures similar to multivesicular bodies (MVBs) along with numerous small vesicles. Both the viral envelope and the small vesicles contained viral glycoproteins and CD63, a marker for MVBs (24,25). Furthermore, we show that MVBs serve to export both the virions and the small vesicles (exosomes) by fusion of the limiting membrane of the MVB with the plasma membrane.

## Results

### HHV-6 envelope glycoprotein B accumulates in a perinuclear cytoplasmic compartment characterized by the presence of CD63

**Figure 1:**
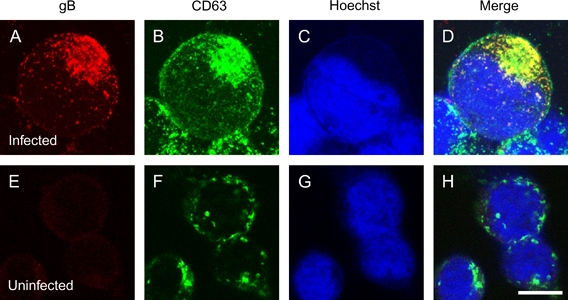
Colocalization of gB and CD63 in HHV-6-infected and uninfected HSB-2 cells A–D) HSB-2 cells infected with HHV-6A by cell-to-cell contact and fixed at 4-day post-infection. E–H) Uninfected cells. The cells were stained with antibodies against gB (A and E) and CD63 (B and F) and with Hoechst 33258 (C and G). The merged panel shows the colocalization of gB with CD63 (D or H). Single sections were shown in this study. Scale bar: 10 μm.

To study the maturation pathway of HHV-6, we first investigated the localization of the HHV-6 envelope glycoprotein, glycoprotein B (gB), which is expressed at the late phase of infection, by fluorescence microscopy. Confocal microscopy of HSB-2 cells infected with HHV-6 (Figure 1A–D) showed that gB and CD63 appeared to colocalize to the same cellular compartment. Nuclear staining (Hoechst) indicated that the compartment was in the cytoplasm of the infected cells (Figure 1C,D). Notably, in uninfected cells, CD63 localized to punctate bodies with a different distribution from that seen in infected cells (Figure 1E–H).

### Electron microscopic examination of HHV-6-infected cells

**Figure 2:**
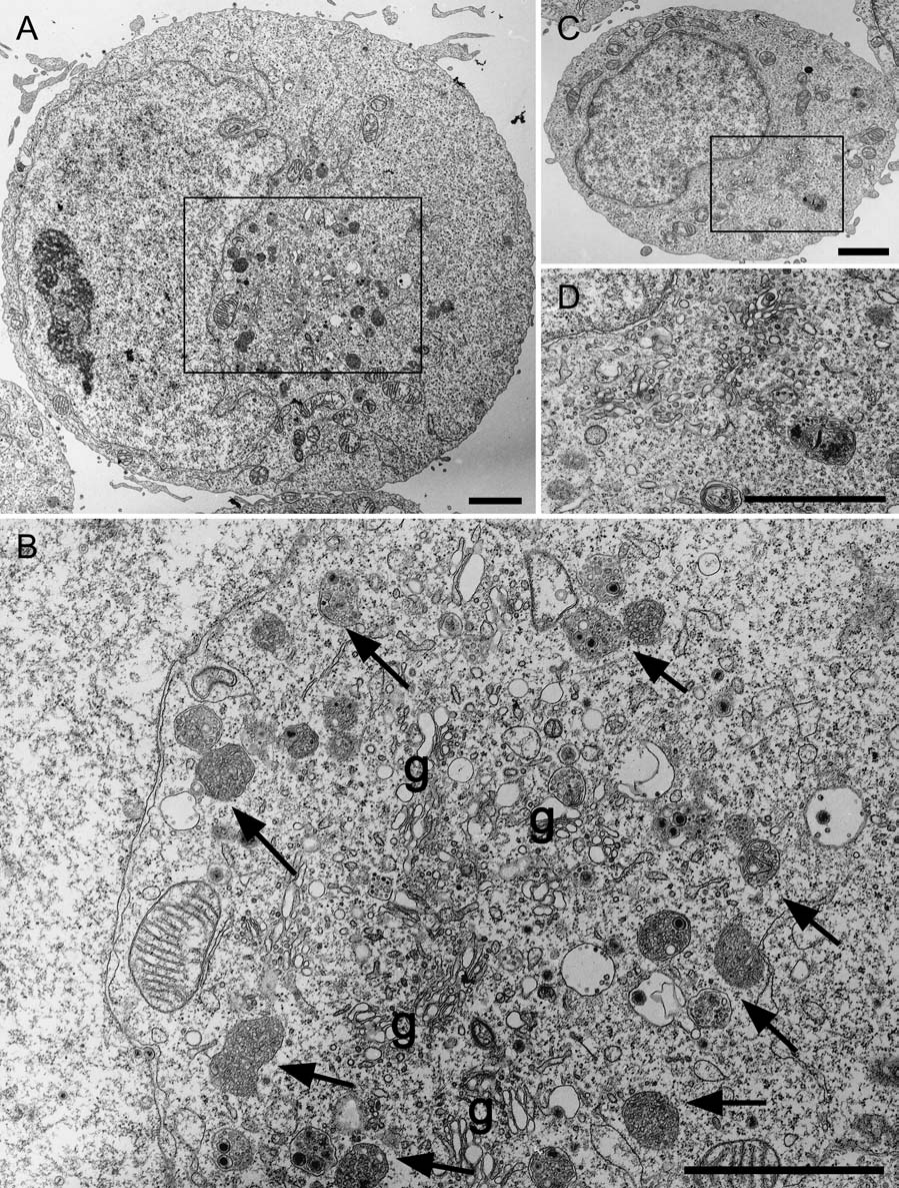
Morphological features of HHV-6-infected HSB-2 cells Ultrathin sections of Epon-embedded cells. Low (A and C) and high [(B) is boxed area in (A) and (D) is boxed area in (C)] power views of HHV6-infected (A and B) and uninfected (C and D) cells. Arrows indicate MVB-like membrane structures around several profiles of the Golgi apparatus (g). Scale bars: 2 μm.

To clarify the intracellular compartment precisely, we examined HHV-6-infected HSB-2 cells by electron microscopy (EM) using ultrathin sections of Epon-embedded cells (Figure 2). As shown in Figure 2, HSB-2 cells infected with HHV-6 were greatly enlarged (Figure 2A) compared with uninfected cells (Figure 2C), indicating that the HHV-6 infection caused ballooning of the cells. Remarkably, many membranous organelles and vacuoles with or without mature virions were observed in the juxtanuclear region of the HHV-6-infected cells (Figure 2A,B) but not in that of the uninfected cells (Figure 2C,D). Indeed, many MVB-like vacuoles were observed in this region (arrows in Figure 2B). Moreover, this perinuclear compartment also contained many Golgi apparatuses (Figure 2B).

The morphometric analyses were performed as described in *Materials and Methods*. The volume density (per cent volume) of various endosomal/lysosomal structures including MVBs in uninfected cells was 2.20 ± 0.26% (mean ± SEM). In contrast, the volume density of such structures in infected cell was 2.7 times larger than that in uninfected cells (p < 0.001, Student's *t*-test) and was 5.93 ± 0.65%. These results indicate that HHV-6 infection induces a special compartment, which is a massive accumulation of membranous organelles including Golgi apparatuses and MVB-like vacuoles, some of which contain virion.

### Primary envelopment and nuclear egress of HHV-6 capsids and AL formation in the infected cells

**Figure 3:**
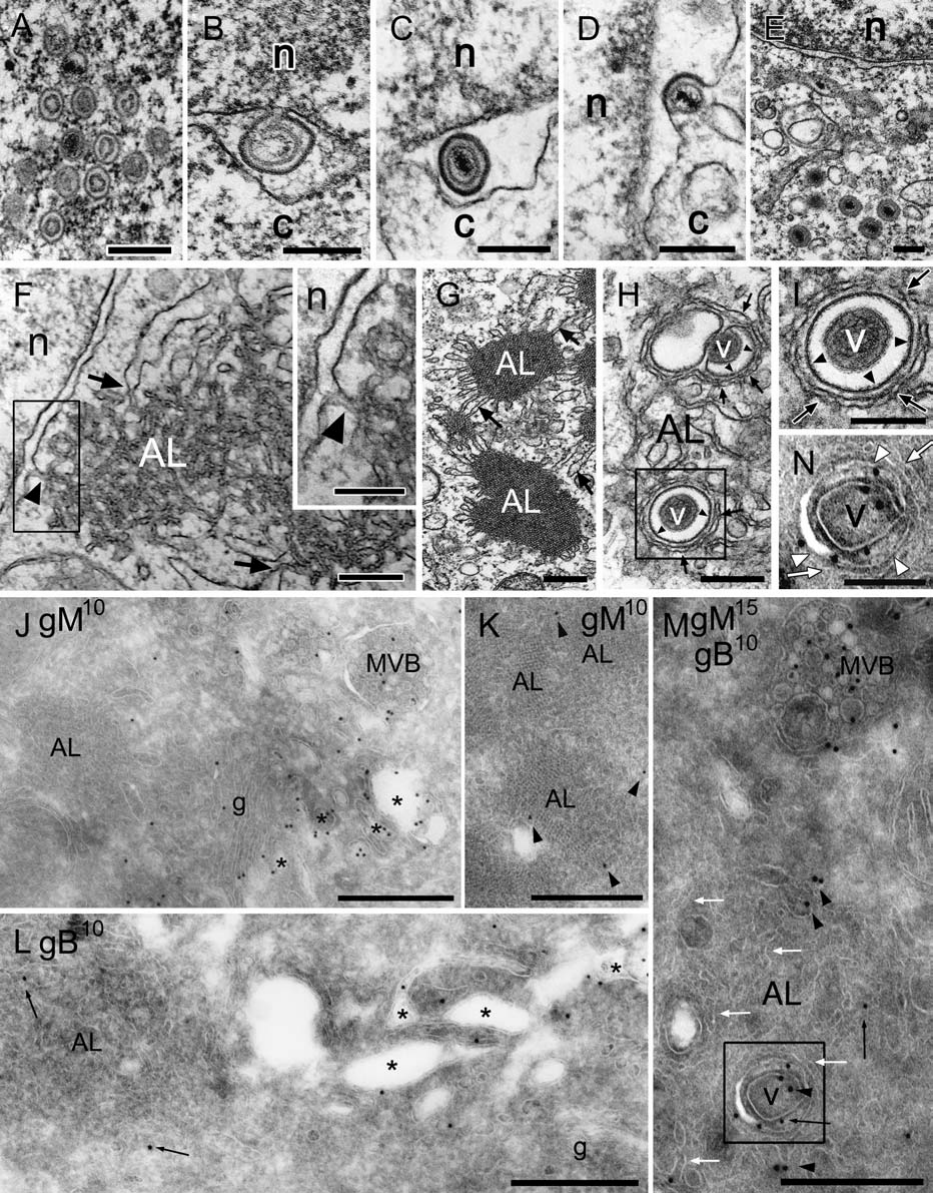
Electron micrographs showing the primary envelopment and nuclear egress of HHV-6 capsids and the formation of AL in HHV-6-infected cells A–I) Ultrathin sections of Epon-embedded cells. J–N) Ultrathin cryosections. A–E) Translocation of capsids from the nucleus (n) to the cytoplasm (c). Intranuclear capsids (A), budding of a capsid from the inner nuclear membrane (primary envelopment) (B), a primary enveloped virion in the cisternal space of the nuclear membrane (C), fusion of primary envelope with the outer nuclear membrane (D) and translocation of the capsids into the cytosol (E). F–I) AL were clearly detected near the nuclear membrane of an infected cell (F). The boxed area in (F) is shown in the inset. The outer membrane of the nuclear envelope was closely associated with the membrane of AL [arrowheads in (F) and the inset in (F)]. Membrane association was also seen between AL and the rough endoplasmic reticulum [arrows in (F and G)]. Note that vesicular or tubular membranes that enwrapped or surrounded virions [arrowheads in (H and I): the boxed area in (H)] were distinct from the membranes of AL cisternae [arrows in (H and I)]. Immunolabeling of gM (J and K) or gB (L) (10 nm) and double immunolabeling of gM (15 nm) and gB (10 nm) (M) on ultrathin cryosections. Gold particles indicating gM- and/or gB-labeled virions (v) and small vesicles located in tubulo-vacuoles (asterisks) in the Golgi complex (g) and MVB but not in AL in the cells. Arrowheads indicate gM and arrows indicate gB (M and N). The boxed area in (M) is shown in (N). AL cisternae are indicated by white arrows. Gold particles indicating gM (black arrowheads) and gB (black arrows) were localized in a virion and the membrane of the vacuole containing the virion [the boxed area in (M) and white arrowheads in (N)] but not in AL cisternae surrounding the vacuole [white arrows in (N)]. Scale bars: 0.3 μm (A–H), 0.2 μm [inset in (F, I and N)] and 0.5 μm (J–M).

To examine the site of virion assembly, we first focused on virion budding in the nuclear membrane by EM using ultrathin sections of Epon-embedded cells (Figure 3A–E). The ultrastructural examination revealed numerous capsids containing DNA in the nucleus (Figure 3A). As reported previously (22,26–29), the primary envelopment of nucleocapsids was observed to occur by budding through the inner leaflet of the nuclear membrane into the perinuclear cisterna. These nucleocapsids were observed in intimate contact with the inner nuclear membrane and were bordered by a sharply delineated rim of electron-dense membrane (Figure 3B). The nucleocapsid was surrounded by a smooth envelope, representing the primary envelope of the virion, and remained in the perinuclear cisterna until it was released into the cytosol by fusion between the primary viral envelope and the outer nuclear membrane, as reported for other herpesviruses (Figure 3C–E) (26–29).

By EM using ultrathin sections of Epon-embedded cells (Figure 3F–I), AL-like structures were observed adjacent to the outer nuclear membrane (Figure 3F) or associated with it (Figure 3F,G) or with rough endoplasmic reticulum (Figure 3F,G), and a few vacuoles were found inside the AL structures (Figure 3H). Occasionally, a few virions were found inside the vacuoles in the AL structures, as reported previously (22); however, the membranes in which the virions were enwrapped or surrounded were not derived from AL because the crescent-shaped vacuole into which a virion was budding and the round vacuole that contained an enveloped capsid inside the AL structure were further enwrapped by AL membrane (Figure 3H,I). Immunolabeling of ultrathin cryosections showed that glycoproteins, gB and glycoprotein M (gM) were scarcely present on the AL, while they were mainly found in vacuoles near the Golgi apparatus, as described later (Figure 3J–N). Remarkably, gB and gM were abundant on the membrane of the vacuoles enwrapping virions that were observed inside the AL structure (white arrowheads in Figure 3N), but not on the membrane of the AL itself (white arrows in Figure 3M,N), suggesting that the origin of the membrane that contained or surrounded the virions was distinct from that of the AL. These results indicated that the membrane of the vacuoles observed in the AL structures might be associated with the TGN, as described later.

### HHV-6 virion budding occurs at TGN- or post-TGN-derived vacuoles

**Figure 4:**
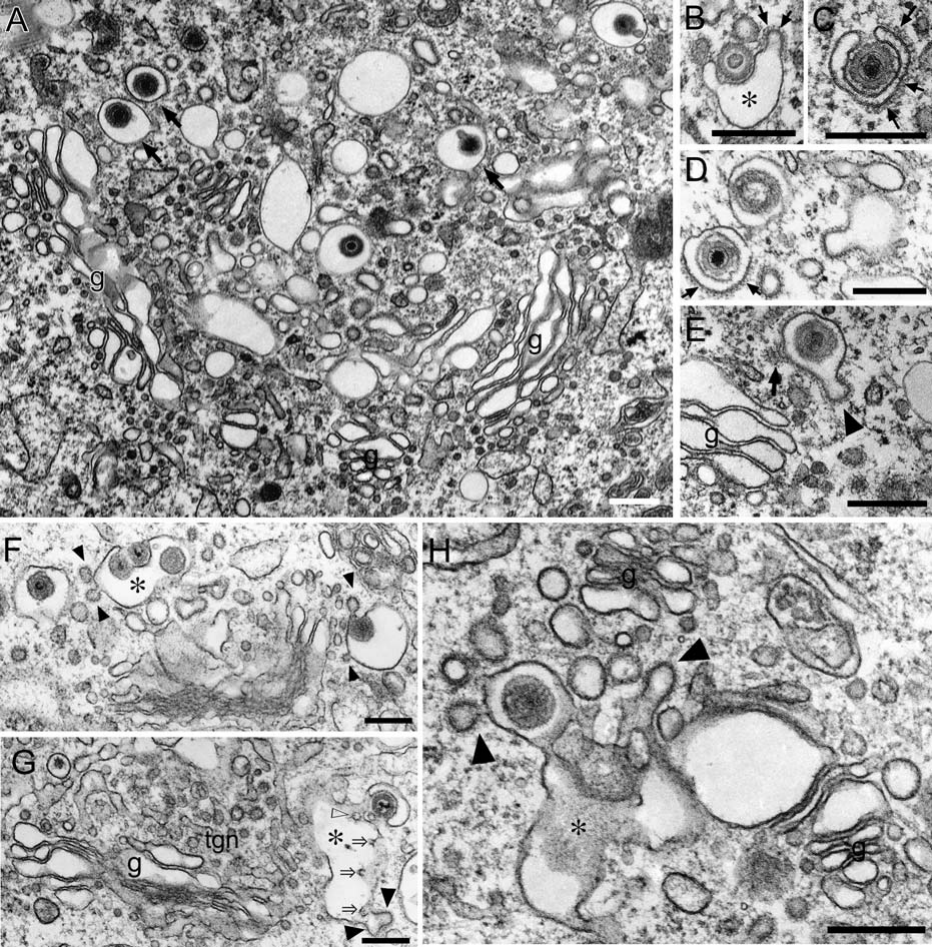
Electron microscopic detection of secondary envelopment Ultrathin sections of Epon-embedded cells. A) Enveloped virions were detected in vacuoles near the Golgi complex (g), and such vacuoles possessed clathrin-coated membrane domains that frequently formed a bud. B–F) Numerous capsids in the cytoplasm close to very dense membrane domains of vacuoles that resembled secondary envelope (B and F) were enwrapped by the membrane to form inward blebs or buds into the vacuolar space [asterisks in (B and F)] and eventually appeared as enveloped virions within the space (D–F). Small arrows in (B–D) indicate clathrin-coated membrane domains. Arrowheads indicate clathrin-coated vesicles budding from the vacuoles containing enveloped virions (E and F). An arrow in (E) indicates part of a polygonal network showing the ‘honeycomb pattern’ formed by clathrin. G and H) Large tubulo-vacuoles (asterisks) were located in or near the TGN (tgn), and they often contained enveloped virions, and clathrin-coated vesicles (arrowheads) budding from such tubulo-vacuoles were observed. Moreover, inwardly budding profiles of such tubulo-vacuoles [open arrows in (G)] and internal vesicles [open arrowhead in (G)] within them were also observed. Scale bars: 0.3 μm.

It has been reported that the final envelopment of HHV-6 may occur at the AL or at the *cis* side of the Golgi complex (22). More recently, the final envelopment was shown to occur in cytoplasmic vesicles (23). Because the AL itself seems not to be the final envelopment site of HHV-6, as described above, we further examined the virus maturation pathway in detail by EM using ultrathin sections of Epon-embedded cells (Figure 4). Numerous capsids, either already enveloped or becoming enveloped by budding into vacuoles, were found near the Golgi complex (Figure 4A). Immature virions were also observed juxtaposed with and partly enveloped by the very dense vacuolar membrane (Figure 4B,C). These vacuoles contained clathrin-coated membrane domains (small arrows in Figure 4B–D), and clathrin-coated vesicles were seen budding from the vacuoles (arrowheads in Figure 4E–H). Large tubulo-vacuoles, some of which contained enveloped virions, were located in or near the TGN and often contained enveloped virions (asterisks in Figure 4F–H), and clathrin-coated vesicles budding from the vacuoles were readily observed. Because clathrin-coated membranes bud from the TGN (30), the membrane of the vacuoles into which the virions budded must have been derived from the TGN, indicating that the cytoplasmic envelopment of HHV-6 takes place at TGN-derived membrane.

**Figure 5:**
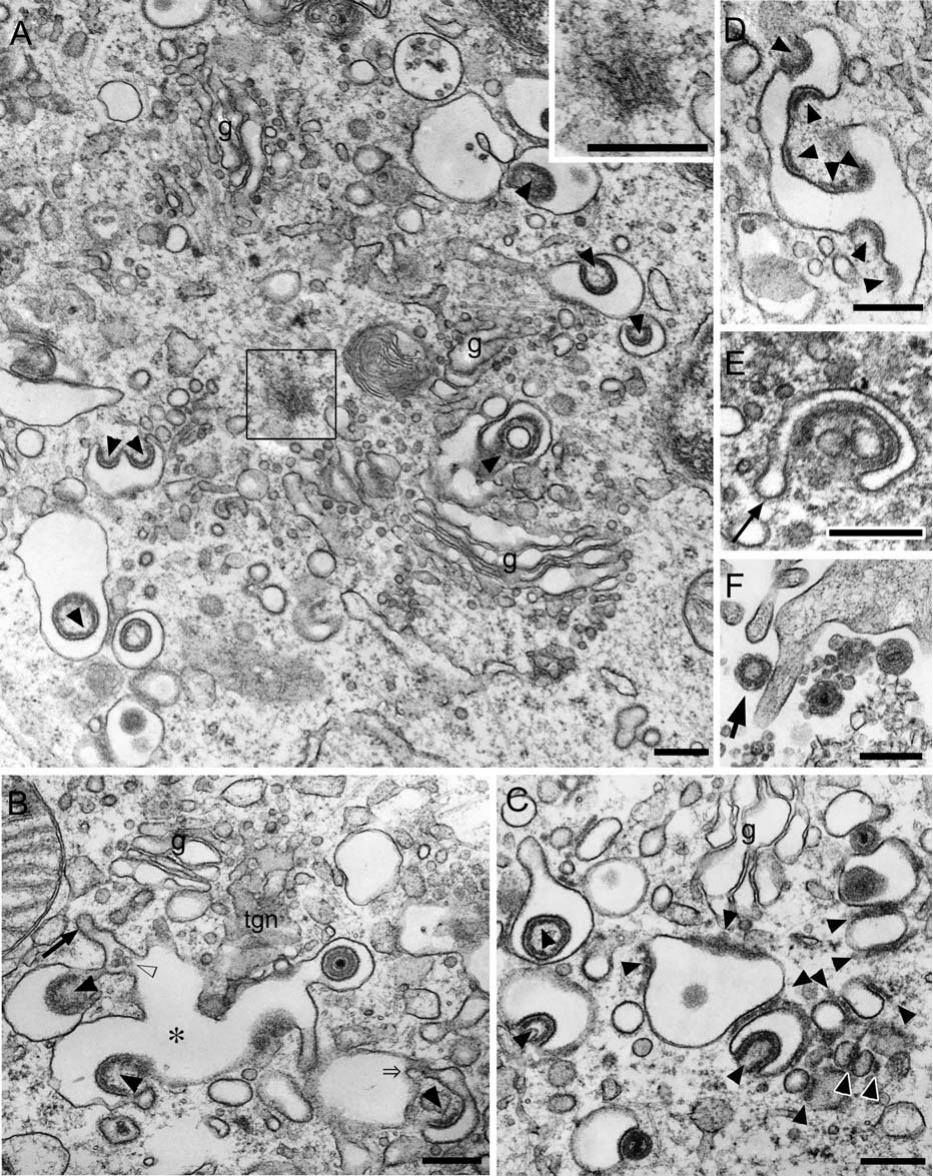
Accumulation of tegument-like materials along the cytosolic face of the TGN-derived vacuoles Ultrathin sections of Epon-embedded cells. A) Numerous vacuoles were observed around the Golgi apparatus (g), and their membrane domains were modified with electron-dense tegument-like materials (arrowheads). Note the microtubule-organizing center (boxed area and the inset). B) Large tubulo-vacuoles (asterisk) were located in or near the TGN (tgn) and often contained enveloped virions, while clathrin-coated vesicles (arrow) budding from such tubulo-vacuoles were observed. Arrowheads indicate electron-dense materials resembling tegument. Moreover, inwardly budding profiles of the membrane of such tubulo-vacuoles (open arrow) with internal vesicles (open arrowhead) were observed. C and D) Examples of vacuoles with multiple membrane domains decorated with electron-dense tegument-like materials (arrowheads). E) An example of the vacuoles with budding clathrin-coated vesicles and multiple membrane domains with electron-dense tegument-like materials (arrow). F) L-particle (arrow) was found near plasma membrane. Scale bars: 0.3 μm.

**Figure 6:**
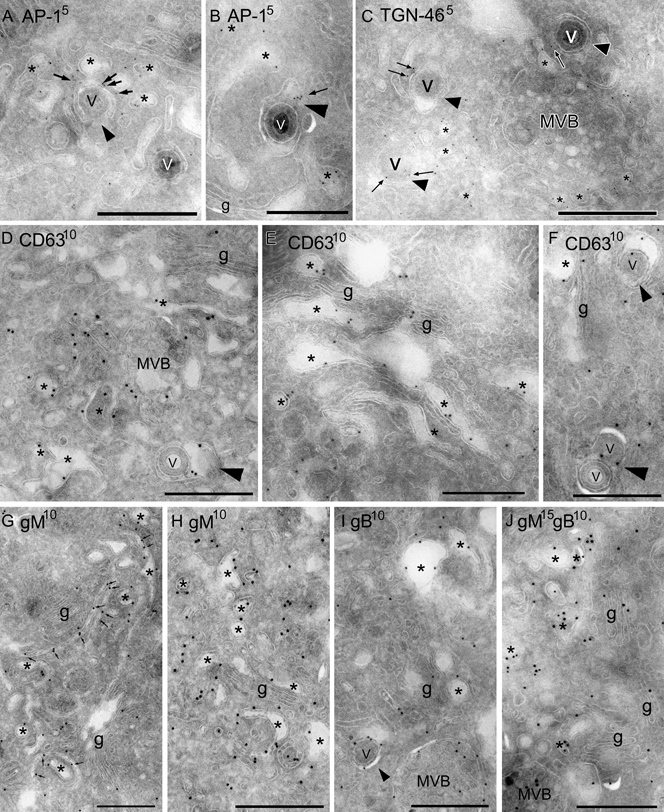
Immunocytochemical detection of AP-1, CD63, gM and gB around the TGN in HHV6A-infected cells Immunogold labeling showing AP-1 (A and B) (5 nm), TGN-46 (C) (5 nm), CD63 (D–F) (10 nm), gM (G and H) (10 nm) or gB (I) (10 nm) or double labeling indicating gM (15 nm) and gB (10 nm) (J) in HHV6A-infected cells on ultrathin cryosections. A and B) Positive signals for AP-1 were localized to vacuoles closely associated with the TGN (asterisks). Note that tubulo-vacuoles containing or incorporating virions (v) (arrowheads) were also immunopositive for AP-1 (arrows). C) Positive signals for TGN-46 are localized to small vacuoles (asterisks) and (tubulo-)vacuoles containing virions (v) (arrowheads) but not in an MVB. D–F) Positive CD63 signals were localized not only to MVBs but were also on (tubulo-)vacuolar structures associated with the TGN (asterisks). Vacuoles that were labeled with immunogold particles for CD63 frequently had incorporated virions (v) (arrowheads). G–I) Positive gM (G and H) or gB (I) signals were seen on tubulo-vacuolar structures (arrows) or vacuoles (asterisks) closely associated with the TGN. Note that a vacuole incorporating a virion (v) (an arrowhead) was heavily labeled with immunogold particles for gB (I). gB signals were also localized to MVBs. J) Vacuoles closely associated with the TGN were often co-labeled with gM (15 nm) and gB (10 nm) (asterisks). g, Golgi complex. Scale bars: 0.5 μm.

Furthermore, by EM observation using ultrathin sections of Epon-embedded cells, we observed electron-dense materials adhered to the cytosolic face of the concave surface of the TGN-derived vacuoles for cytoplasmic envelopment (Figure 5), and interestingly, these materials were also found at several positions on the vacuoles (Figure 5B–D). Because these materials were unique in infected cells, they were thought to be viral tegument-like materials, as described elsewhere (11,27). In our observations, the tegument-like materials accumulated specifically along such vacuoles. In some cases, the addition of enormous amounts of tegument materials was observed as reported in the other herpesviruses (11,27), possibly indicating that it results in the formation of L-particles that are enveloped viral particles without capsids (an arrow in Figure 6F) (11,27).

In addition, immunolabeling of ultrathin cryosections showed that adaptor protein (AP)-1, which is involved in the assembly of clathrin-coated vesicles originating from the TGN (31), was localized to vacuoles closely associated with the TGN and to tubulo-vacuoles (arrows in Figure 6A,B) containing virions (arrowheads in Figure 6A,B). In addition, anti-TGN46 antibody (Ab), which is generally used as a marker of TGN, also labeled tubulo-vacuoles containing virions (arrows in Figure 6C). As expected, the viral envelope glycoproteins gM and gB were also detected on the membrane of vacuoles in the vicinity of the Golgi apparatus (Figure 6G–J). Interestingly, CD63, which is usually present in late endosomes/MVBs (32), in infected cells was localized to tubulo-vacuolar structures that were closely associated with or composed of the TGN (asterisks in Figure 6D–F), to the membrane of the vacuoles containing virions (arrowheads in Figure 6D,F).

Taken together, these findings indicated that the membrane of the vacuoles into which the virions bud has characteristics of both the TGN and endosomes, suggesting that these vacuoles are post-TGN derived. Indeed, inward budding profiles of the membrane of such tubulo-vacuoles (open arrows in Figures 4G and 5B) containing internal vesicles (open arrowhead in Figure 5B) were sometimes observed.

### HHV-6 infection induces the formation of MVB-like vacuoles, which might be derived from post-TGN-derived vacuoles

**Figure 7:**
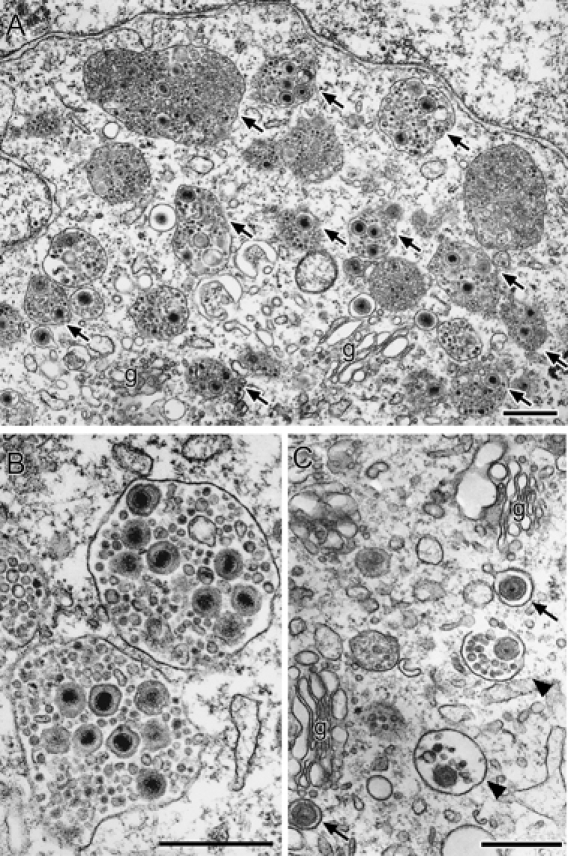
Formation of MVBs containing enveloped virions in the cytoplasm of HHV6-infected cells Ultrathin sections of Epon-embedded cells. A) A massive accumulation of MVBs in the perinuclear region of an infected cell. The MVBs often contain enveloped virions (arrows). g, Golgi apparatus. B) Examples of MVBs with numerous intact enveloped virions and internal vesicles. C) Small vacuoles (arrows) containing virions adjacent to Golgi apparatus (g) and larger MVB-like vacuoles containing virions and internal small vesicles (arrowheads). Scale bars: 0.5 μm.

As shown in Figure 2, HHV-6-infected HSB-2 cells formed many MVBs that contained mature virions and small vesicles (Figure 2A,B). By careful observation, by EM using ultrathin sections of Epon-embedded cells (Figure 7A,B), we noticed that MVBs often contained intact virions. MVBs function as intermediates in the degradation of proteins internalized from the cell surface or sorted from the TGN (33). Indeed, a mature virion in a small vacuole (arrow in Figure 7C) was observed near the Golgi apparatus and TGN, and other larger vacuoles containing both virions and internal vesicles were found close to this small vacuole (arrowheads in Figure 7C), indicating that the MVB-like vacuoles might have been derived from TGN-associated vacuoles. Occasionally, in addition to virions, several internal small vesicles were also observed within a single vacuole (Figure 7C). These structures were probably intermediates between the TGN-associated vacuoles and the mature MVBs. These results are consistent with a scenario in which a small vacuole containing virions gradually expands and forms internal vesicles, resulting in the formation of MVBs.

**Figure 8:**
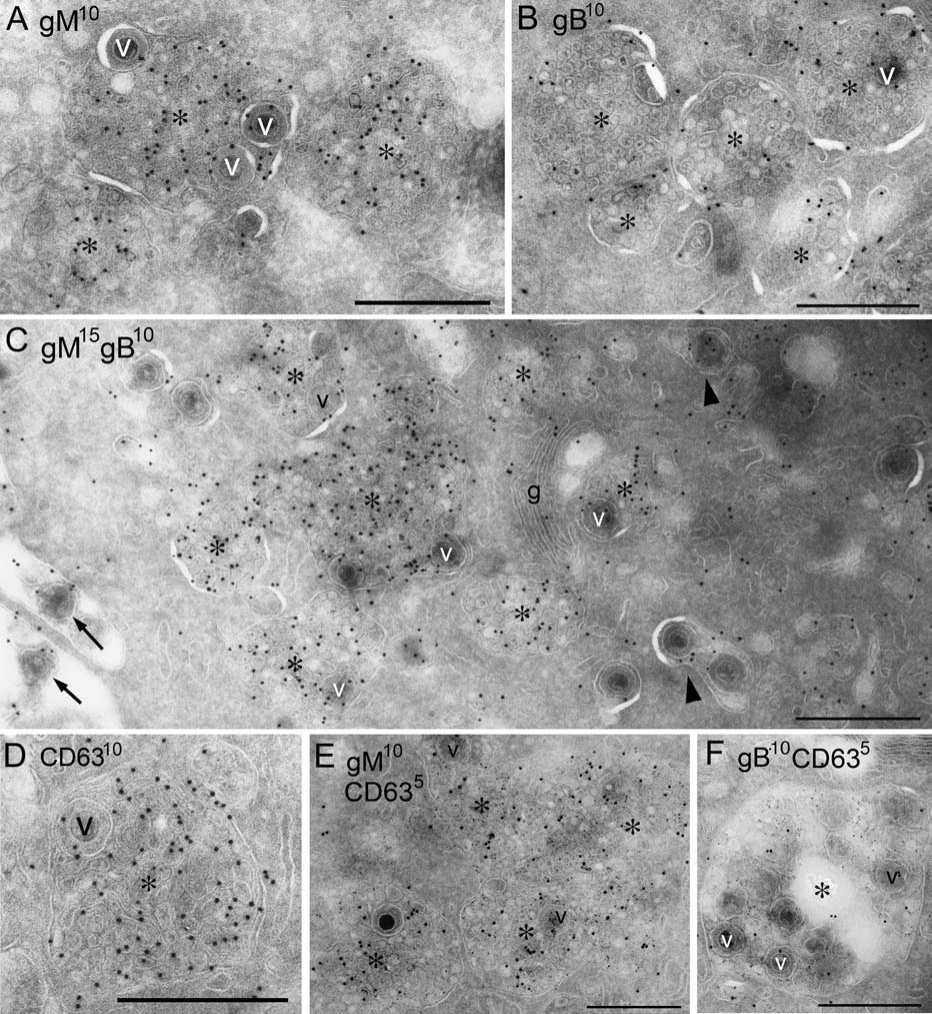
Accumulation of gB, gM and CD63 in MVBs Immunogold labeling on ultrathin cryosections of HHV6A-infected cells showing gM (A) (10 nm), gB (B) or CD63 (D) (10 nm) or double labeling indicating gM (15 nm) and gB (10 nm) (C) or CD63 (5 nm) and gM (10 nm) (E) or gB (10 nm) (F). A–C) Immunogold particles indicating gM and/or gB were localized to both the internal vesicles of MVBs (asterisks) and the virions (v) within them. Note that extracellular virions (arrows), smaller vacuoles that contained only virions (arrowheads) and the Golgi apparatus (g) were also immunopositive for gM and gB. D) CD63 was localized to the internal vesicles of the MVBs (asterisk) and the virions (v) within them. E and F) Immunogold particles indicating gM (E) or gB (F) with CD63 were colocalized to the internal vesicles of the MVBs (asterisks) and the virions (v) within them. Scale bars: 0.5 μm.

Immunoelectron microscopy using ultrathin cryosections revealed that the virion-containing vacuoles were strongly labeled with the anti-CD63 Ab (asterisks in Figure 8D), confirming that the vacuoles represented MVBs. As described previously, gM and gB were detected on tubulo-vacuolar structures associated with the TGN (Figures 6G–J and 8C). Massive gB or gM staining was also found on the MVBs (Figure 8A–C, E and F). Furthermore, not only gB and gM but also CD63 were incorporated into virions (Figures 6F and 8D–F). Most gB or gM staining was associated with the MVBs and TGN of infected cells, consistent with the results shown in Figure 1.

### HHV-6 egress occurs through the MVBs by an exosomal release pathway

**Figure 9:**
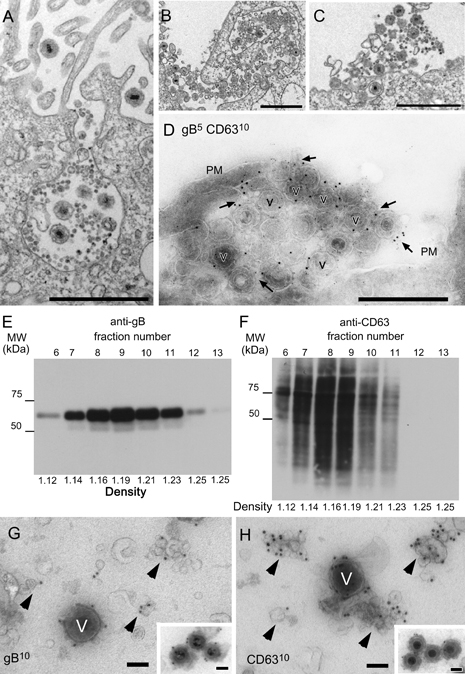
Exosomes contribute to the release of enveloped virions A–C) Ultrathin sections of Epon-embedded cells. MVBs were exocytosed from the cell surface, and numerous virions along with small internal vesicles were released from the cells. Released virions and exosome-like small internal vesicles were detected in the vicinity of the cell membrane (B and C). D) Double immunogold labeling of gB (5 nm) and CD63 (10 nm) in HHV6A-infected cells on ultrathin cryosections. Some virions (v) and exosomes (arrows) were positive for both gB and CD63. PM, plasma membrane. E–H) Exosome fractions containing virions were collected by sucrose density gradient from the culture medium of HHV-6A-infected T cells and were analyzed by immunoblotting and EM. E and F) Immunoblot analysis with anti-gB (E) or anti-CD63 (F) antibodies of the sucrose density gradient fractions. The same volume of the gradient fraction was used for all blots. Densities of the fractions are listed at the bottom in grams/milliliters. G and H) Immunogold labeling of gB (G) and CD63 (H) in whole-mount virions and exosomes from fraction 9 in panels (E) or (F). Gold particles showing gB (G) and CD63 (H) labeling of exosomes (arrowheads) and virions (v). Insets in (G and H) showed immunogold labeling of gB and CD63 in purified virions, respectively. Gold particles indicating gB [an inset in (G)] and CD63 [an inset in (H)] were detected in purified virions (v). Scale bars: 1 μm (A–C), 0.5 μm (D), 100 nm [(G and H) and insets in (G and H)].

It has been demonstrated that the internal vesicles in MVBs have three distinct fates (33,34). First, they target incorporated proteins to lysosomes for degradation, a process that requires the direct fusion of MVBs with lysosomes. Second, MVBs can also serve as temporary storage compartments. Third, they can secrete their internal components as a consequence of fusion between their limiting membrane and the plasma membrane. The internal MVB vesicles undergoing this process are designated as exosomes (33–36). Consistent with the hypothesis that MVBs serve to secrete their contents in infected cells (37), when observed by EM using ultrathin sections of Epon-embedded cells, we also noted that mature virions were seen in invaginations or deep pockets of the plasma membrane (Figure 9A,B) and on the surface of the plasma membrane (Figure 9B,C), and these virions were interspersed with small vesicles resembling exosomes (Figure 9C).

To test the hypothesis that the MVBs serve to export virions from infected cells, we performed immunoelectron microscopy using ultrathin cryosections of infected cells. HHV-6 envelope glycoproteins, gB and gM were readily detected on the extracellular virions, but they were not found in the plasma membrane. It is noteworthy that the extracellular virions also contained CD63, and at the same time, gB (Figure 9D) and gM (data not shown) were detected on CD63-positive extracellular small vesicles that were consistent with exosomes in appearance.

To verify that the envelope glycoproteins were expressed on exosomes as well as on virions, the exosomes were isolated from the culture medium of HHV-6-infected T cells. Both gB (Figure 9E) and CD63 (Figure 9F) were detected in the exosome fractions (37) by immunoblotting. The typical characteristics of exosomes, which are cup-shaped membrane vesicles 50–90 nm in diameter (33), were observed in fraction 9 (Figure 9E or F) by whole-mount EM, and this fraction also contained virions approximately 200 nm in diameter, although their frequency was low (Figure 9G,H). Immunogold labeling of whole-mount exosomes showed that some exosomes were stained with gB, confirming that viral glycoproteins were expressed on exosomes (arrowheads in Figure 9G). Remarkably, CD63 was detected on virions as well as on exosomes (arrowheads in Figure 9H). To confirm that CD63 protein is expressed on virions, virions were purified by Histodenz linear gradient and stained with anti-CD63 or gB monoclonal antibody (mAb). Both gB and CD63 were detected on virions (insets in Figure 9G,H).

**Figure 10:**
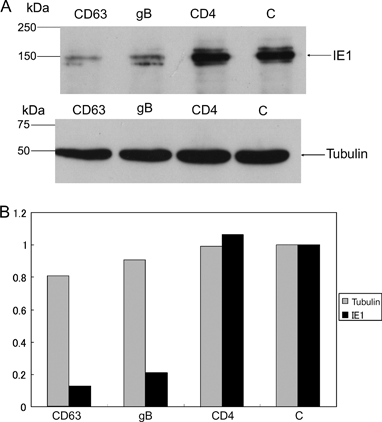
The infectivity of viruses secreted through the exosomal release pathway The supernatants of the medium of HHV-6-infected cells were collected and incubated with mAb against CD63 (CD63), gB (gB) or CD4 (CD4) at 4°C for 16 h. The supernatants were also incubated without mAb (C). Immunoprecipitation of viruses was performed by using mAb-based virus precipitation assay as described in *Materials and Methods*. The viruses non-immunoprecipitated were incubated with HSB-2 cells at 37°C for 1 h, and at 48-h post-incubation, the cells were harvested and lysed. A) Western blot of the lysates was performed with anti-IE1 or tubulin mAb. B) Quantitative analysis of western blot by kodak mi software shows the intensity of the band relative to that of C (without mAb). One of three independent experiments was shown. The mAb for CD4 was used as a negative control. The mAb for HHV-6 immediate early protein (IE1), which is not a viral structural protein, was used for the examination of HHV-6 infectivity, and the mAb for tubulin was used as an internal control of proteins.

Next, to examine that viruses inside MVBs and secreted through the exosomal pathway are infectious, the viruses recovered from medium of infected cells were immunoprecipitated using mAb against CD63, and non-precipitated viruses were analyzed by infectivity assays. As shown in Figure 10, the expression levels of HHV-6 immediate early protein-1 (IE1) were low in lane CD63 as well as lane gB, while they were high in lanes CD4 and C (without mAb), indicating that the infectivity of viruses that were not precipitated with CD63 mAb was reduced compared with that with CD4 mAb used as negative control. These results suggest that the viruses secreted through the exosomal pathway are infectious.

We conclude from these results that in the course of cytoplasmic envelopment, virions acquire an envelope containing CD63 in addition to viral glycoproteins, from TGN- or post-TGN-derived membranes, and that MVBs generated in the course of infection export virions as well as exosomes from the infected cells (Figure 12).

### Intracellular distribution of the Golgi apparatus, MVBs and AL in HHV-6-infected T cells

**Figure 11:**
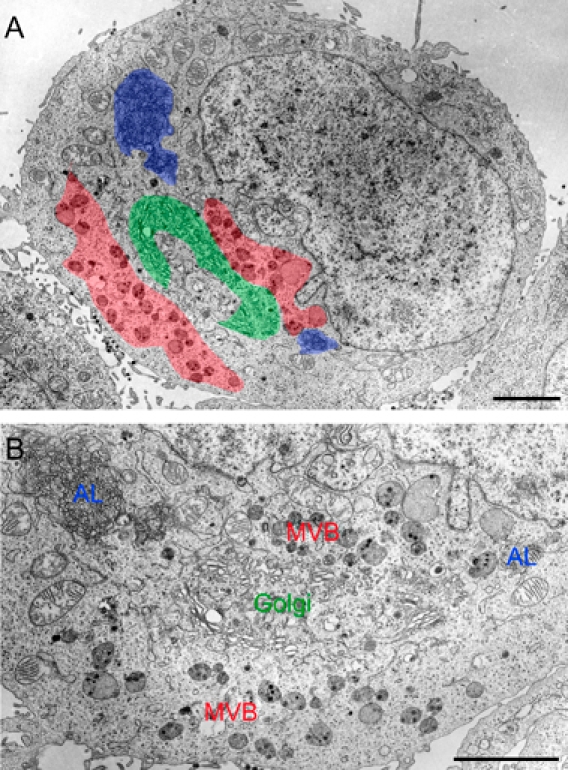
Intracellular distribution of the Golgi apparatus, MVBs and AL in an HHV6A-infected cell Ultrathin sections of Epon-embedded cells. A cluster of Golgi apparatus together with tubulo-vacuolar structures [marked by green color in (A) and Golgi in (B)] was located in the center of surrounding MVBs [marked by red color in (A) and MVB in (B)]. AL [blue in (A) and marked ‘AL’ in (B)] were observed apart from the Golgi apparatus and MVBs. Scale bars: 3 μm.

Our EM findings are summarized in Figure 11, which shows the intracellular distribution of organelles in an HHV-6-infected T cell. A cluster of Golgi apparatuses with tubulo-vacuolar structures was located in the center of the surrounding MVBs (Figure 11A,B). This organization indicates a possible pathway from cytoplasmic immature virions to mature enveloped virions. A cluster of MVBs was located in the outer part of this organization, near the cell surface membrane, from which completed virions, together with small vesicles, were released outside the cell. A structure where final virion assembly and maturation take place is already known as the virion assembly complex (AC) or viral field (17,18,26). In the case of HHV-6, this area containing the Golgi apparatus and MVBs appears to correspond to the AC. In our observations, AL was located in areas that were away from the cluster of Golgi apparatus and MVBs, indicating that AL does not seem to be involved in the AC.

## Discussion

**Figure 12:**
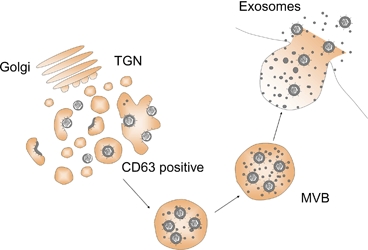
Model of virus maturation and egress An HHV-6 mature virion is first assembled in a TGN- or post-TGN-derived vacuole that expresses CD63 and may later become a large vacuole containing small vesicles, which has similar characteristics to MVBs. The virions are released into the extracellular environment together with small vesicles by the exosomal secretion pathway.

The budding and maturation pathways of HHV-6 have not been well studied and are poorly understood. We undertook this study to address these questions and more generally to analyze the cell biology of HHV-6 assembly and release from infected T cells. To this end, we first analyzed the intracellular localization of the HHV-6 structural components, especially an envelope protein of HHV-6A, gB, at a late stage of HHV-6 infection, by immunofluorescence microscopy. We found that gB was colocalized partially with CD63 in the juxtanuclear area, indicating that it was, at least in part, associated with late endosomes. Therefore, we next studied the intracellular maturation pathway of HHV-6 in more detail by EM and found novel pathways, some of which differed from those of the alphaherpesviruses. We also observed the AL structure in infected cells and found a few mature virions in the AL, as reported previously (22); however, the vesicular or tubular membranes that surrounded the virions appeared to be distinct from the membrane of AL cisternae. Most importantly, our observations showed that the final envelopment of HHV-6 occurred at TGN- or post-TGN-derived membranes. Remarkably, the vacuoles that enwrapped the mature virions contained clathrin-coated membrane domains that frequently formed a bud. This is the first report of this observation for a herpesvirus. This might show that the membrane for HHV-6 budding is derived from the TGN, and we confirmed this idea by performing immunoelectron microscopy using anti-TGN46 Ab. Interestingly, CD63, which is usually considered a marker for late endosomes/MVBs, was also detected on the membrane of vacuoles incorporating a virion and on virions themselves. These results indicated that the virus-wrapping membrane may have characteristics intermediate between those of the TGN and endosomes. Therefore, we concluded that HHV-6 budding occurs not only at TGN-derived membranes but also at post-TGN-derived membranes (Figure 12). Furthermore, we noticed that tegument-like materials specifically accumulated along the membrane of such vacuoles. The addition of enormous amounts of tegument materials as shown in Figure 5 may result in the formation of L-particles, which are enveloped viral particles without capsids but not complete particles (11,27).

Recently, Fraile-Ramos et al. suggested a similar scenario for the final envelopment of HCMV in which the membranes for final envelopment traffic to or from the TGN or in which the virus generates a novel membrane containing CD63 and markers for the TGN because CD63 labeling was seen on the membranes enwrapping virus particles and dense bodies and on extracellular enveloped HCMV (38). However, early endosomes have also been proposed to play a role in HCMV maturation and egress (18).

Our ultrastructural analysis of HHV-6-infected HSB-2 cells also showed the presence of virions in large intracellular vacuoles in the vicinity of the Golgi apparatus. The virus-containing vacuoles had a complex morphology and frequently contained many small vesicles resembling the internal vesicles in MVBs (33,34,36). Immunolabeling of ultrathin cryosections showed that the virus-containing vacuoles had high levels of CD63, a marker of MVBs, and the structure of the limiting membrane appeared to be similar to that of late endosomes, indicating that the vacuoles induced by HHV-6 infection were MVBs.

Interestingly, the MVBs appeared to be newly formed in HSB-2 cells by HHV-6 infection, because they were not seen in uninfected cells, and the cluster of Golgi apparatus was located in the center of surrounding MVBs. Even in our intensive EM observations, no fusion between TGN-associated vacuoles and MVBs or direct HHV-6 budding from an MVB membrane was ever seen. Therefore, our observations shown in Figure 7 indicate that MVBs containing mature virions are derived from post-TGN-derived vacuoles. In addition, no tegument-like materials were seen to accumulate at the MVB membrane. It is unclear why all the machinery needed for HHV-6 assembly is not contained in the MVB membrane. Moreover, we observed the release of enveloped virions together with internal small vesicles into the extracellular milieu upon exocytotic fusion of MVBs with the cell surface, implying an arrangement for the route of enveloped virus egress (Figure 11). In other words, enveloped virions are released by the exosomal pathway. These results indicate that HHV-6 hijacks the cellular machinery normally used for vesicle formation and trafficking, modifying it for virus maturation and secretion. A recent EM analysis of HCMV shows viral particles within MVBs and occasionally budding into MVBs; therefore, MVBs might also be the final budding site of HCMV (19). Furthermore, HCMV glycoproteins, including several viral chemokine receptor-like proteins, are also present in virions and enriched in the virus-wrapping membrane and MVBs and might be incorporated into the viral membrane during the budding step (19,39). Moreover, MVBs and cytoplasmic dense bodies are also observed in HCMV-infected human bone marrow fibroblasts (26).

HIV type 1 (HIV-1) virions are generally thought to assemble at the plasma membrane of infected T cells, and the virions are secreted into the culture medium when the virus-containing vacuoles fuse with the plasma membrane (40). Furthermore, recent studies have suggested that HIV-1 assembles into an internally sequestered CD63-positive plasma membrane domain containing several tetraspanins but not into an endosome of HIV-1-infected macrophages (41,42).

Another characteristic of the TGN-associated vacuoles in our observations was a thick, electron-dense coat that frequently extended over a large proportion of the cytoplasmic region of the vacuole membrane. Because the vacuoles that contained HHV-6 virions contained many small vesicles and had a thinner limiting membrane than that of TGN-associated vacuoles, these vacuoles appear to be classical MVBs.

Immunolabeling of cryosections in this study revealed the viral envelope glycoproteins gM and gB to be located on small vesicles inside MVBs and on exosomes in the culture medium. The HHV-6 envelope is derived from TGN- or post-TGN-derived membrane as described above; however, the internal vesicles are derived from MVB (endosomal) membrane, indicating that the gB and gM proteins expressed on the internal small vesicles may bud from the MVB membrane directly and become incorporated into the small vesicles in a manner similar to that of cellular proteins expressed on the small vesicles. During the course of virus maturation, they would be selectively sorted to the MVB membrane by the cellular pathway of intracellular vesicle trafficking, although the sorting mechanism for the viral proteins is still unknown.

In general, exosomes (37), which are membrane vesicles with a diameter of 40–100 nm, are released from the cell by the fusion of MVBs with the plasma membrane (33–35,43) and contain membrane proteins normally found in late endosomes, such as CD63, as well as major histocompatibility complex molecules (44). This raises the question what are the roles of the exosomes produced in HHV-6-infected T cells? Recently, Wiley and Gummuluru reported that immature dendritic cell-derived exosomes could mediate HIV-1 transinfection of T cells (45). Several reports have shown T-cell activation by exosomes. Exosomes secreted by antigen-presenting cells have the ability to stimulate T-cell proliferation in T cells (37). Dendritic cell-derived exosomes induce antigen-specific naïve CD4+ T-cell activation *in vivo* (46). The presence of intercellular adhesion molecule 1 in exosomes from mature DCs may be essential for an indirect stimulation of T cells (47). The incubation of free exosomes with DCs results in the highly efficient stimulation of specific T cells (48). Inasmuch as HHV-6 replicates well in stimulated T cells, it is conceivable that the exosomes derived from HHV-6-infected T cells may induce T-cell activation for virus replication. Another hypothesis for the role of exosomes centers on the evidence that HHV-6 replicates more efficiently after virus spreading by direct cell-to-cell contact than after cell-free spread. It is conceivable that in T cells, the viral glycoproteins and cellular proteins expressed on exosomes may interact to form a ‘virological synapse’ to promote the efficient spreading of virus from infected to uninfected cells (49–52).

In conclusion, our results indicate an important role for MVB formation in the biogenesis of HHV-6, and this is a key finding toward elucidating the mechanism of membrane fission during HHV-6 budding and MVB vesicle formation that is distinct from previously well-studied membrane fission events. Further investigation is needed to determine the vesicular and molecular signals and proteins exploited by HHV-6 in this process.

## Materials and Methods

### Cells and viruses

The HSB-2 T-cell line was cultured in RPMI-1640 medium supplemented with 8% fetal bovine serum. The HHV-6A strain GS was propagated and titrated in HSB-2 cells. HHV-6 cell-free virus was prepared as described elsewhere (53).

### Virion purification

Supernatants containing the virions from infected cells were collected (spun at 2500 × ***g*** at 4°C for 15 min), and the viruses were precipitated with 20% polyethylene glycol (molecular mass 20 kDa) in the presence of NaCl (0.9%). The precipitates were resuspended, layered over a 5–50% Histodenz (Sigma) linear gradient and spun for 1 h at 120 000 × ***g***. The fractions were collected from the bottom, and the virus-containing fractions were determined by a polymerase chain reaction analysis of the viral DNA.

### Antibodies

The OHV-1 mAb against gB (54) and AIE1 mAb against HHV-6 immediate early-1 protein (IE-1) (55) were described previously. Polyclonal antibodies against gB or gM were made by five immunizations of rabbits. The gB antigen, designated AgB-c, was expressed as a glutathione S-transferase fusion protein. Specifically, gB regions amplified from HHV6 DNA by primers AgB2232bamF (5′-accggatccacacctagtgttaaggatgttg) and AgBsalR (accgtcgactcacgcttcttctacatttac) (underlining indicates restriction enzyme site) were inserted into the prokaryotic expression vector, pGEX-4T (GE Healthcare Bio-Sciences) at the *Bam*HI and *Sal*I sites. The chimeric protein was expressed in *Escherichia coli* and purified with glutathione Sepharose 4B (GE Healthcare). The antiserum specific for HHV-6 gM was prepared by immunizing the rabbit with a synthetic peptide, CLVNTESSSLMDENE. The mAbs against CD63 (clone: CLB-Gran1/2, 435; Sanquin), CD4 (clone: 34930; R&D systems, Inc.), γ subunit of AP-1 (clone: 100/3; Sigma) and α-tubulin (clone: B-5-1-2; Sigma) were purchased. Polyclonal Ab for TGN46 (AbD Serotec) was also purchased. The secondary Ab was the Alexa Fluor 488- or 594-conjugated F(ab′)2 fragment of goat anti-mouse or rabbit immunoglobulin G (IgG) (Invitrogen).

### Immunoblotting

Immunoblotting was performed as described previously (53,56).

### Immunofluorescence assay

The immunofluorescence assay was performed as described previously (53). Specific immunofluorescence was observed with a confocal laser-scanning microscope, Leica DMIRE2 (Leica Microsystems).

### Electron microscopy

Cells were fixed in 2% glutaraldehyde–2% paraformaldehyde (PA) buffered with 0.1 m phosphate buffer (PB) (pH 7.2), postfixed with 1% OsO4 buffered with 0.1 m PB (pH 7.2) and embedded in Epon 812. Ultrathin sections were cut with an ultramicrotome (Ultracut N; Reichert-Nissei or UC6; Leica Microsystems) and observed with a Hitachi H-7100 or H-7650 electron microscope.

### Immunoelectron microscopy using ultrathin cryosections

Ultrathin cryosections were prepared as reported elsewhere (57,58). Briefly, cells were fixed with 4% PA buffered with 0.1 m PB (pH 7.2) for 2 h at room temperature and embedded in 12% gelatin in 0.1 m PB (pH 7.2). Small blocks were rotated in 2.3 m sucrose in PB overnight at 4°C and quickly plunged into liquid nitrogen. Sections approximately 60 nm thick were cut with a Leica UC6/FC6 ultramicrotome and picked up with a 1:1 mixture of 2% methylcellulose and 2.3 m sucrose. Immunostaining was performed according to Raposo et al. (37). The sections were reacted overnight at 4°C with rabbit or mouse anti-gB (1:100), rabbit anti-gM (1:100), mouse anti-γ subunit of AP-1 (1:10), sheep anti-TGN-46 (1:20) or mouse anti-CD63 (1:10) and then for 1 h at room temperature with goat anti-rabbit or mouse IgG conjugated with 5- or 10-nm colloidal gold particles (GE Healthcare) or anti-sheep IgG conjugated with 5-nm colloidal gold particles (British Biocell International) and examined with a Hitachi H-7100 electron microscope. For control experiments, ultrathin sections were reacted only with the gold-particle-conjugated secondary Ab.

### Exosome isolation

Exosomes were collected from the cell culture medium by differential centrifugation as described (37) with several modifications. The cells were pelleted by centrifugation for 10 min at 500 × ***g***. The supernatant fluids were collected and subjected to sequential centrifugation once for 30 min at 5000 × ***g*** and once for 60 min at 70 000 × ***g***. The exosomes pelleted by the final centrifugation step were resuspended in PBS, filtered through a 0.2-μm filter (PALL), spun again for 60 min at 70 000 × ***g*** and resuspended in 2 mL of 2.5 m sucrose and 20 mm HEPES/NaOH, pH 7.4. A linear sucrose gradient (2 to 0.25 m sucrose and 20 mm HEPES/NaOH, pH 7.4) was layered on top of the exosome suspension and spun for 16 h at 110 000 × ***g***, after which 500-μL fractions were collected from the top of the tube. The fractions were analyzed by immunoblotting and EM.

### EM of whole-mounted exosomes and virions

EM of the isolated fractions with or without immunogold labeling was performed according to the method of Raposo et al. (37). Briefly, the 100 000 × ***g*** exosome-containing pellet was resuspended in 2% PA buffered with 0.1 m PB (pH 7.2). To adsorb exosomes or virions on electron microscope grids, 5 μL of the resuspended pellet was loaded onto Formvar–carbon-coated grids. Thereafter, the immunogold labeling of vesicle fractions was performed using the method for ultrathin cryosections described above. After immunolabeling, the samples were washed in distilled water, stained for 5 min with uranyl oxalate, pH 7.0, washed again, embedded in a mixture of 1.8% methylcellulose and 0.4% uranyl acetate, pH 4.0, at 4°C, air-dried and observed with a Hitachi H-7100 electron microscope. For control experiments, samples were directly incubated with the secondary Ab without pretreatment with the primary antibodies.

### Morphometry

Morphometric analyses were performed according to the method of Koike et al. (59) with minor modifications. Electron micrographs of the HHV-6-infected HSB-2 cells (*n* = 49) and uninfected cells (*n* = 54) were randomly taken using Hitachi H-7650 electron microscope with a final magnification of ×12 500. Three Epon blocks from each sample were used. After printing, we estimated the cytoplasmic (perikaryal) volume fraction of lysosomal structures including endosomes, MVBs and dense bodies by point counting using a double-lattice test system of 1.5-cm spacing. The volume density (Vv) of each lysosomal structure was expressed as the per cent volume: Vv = (Pi/Pt) × 100 (%), where Pi is the number of points falling on each lysosomal structure and Pt is the number of points falling on the perikarya of cells. The data are expressed as mean ± standard error of the mean. The differences between infected and uninfected cells were evaluated by Student's *t*-test.

### Virus immunoprecipitation assay

Virus immunoprecipitation assay was performed with several modifications as described previously (60). Viruses recovered from the medium of HHV-6-infected cells were incubated with each mAb in a total volume of 500 μL at 4°C for 16 h. Pansorbin cells (formalin-fixed *Staphylococcus aureus* strain Cowan, 25 μL; Calbiochem) were incubated with PBS–3% BSA or with rabbit anti-mouse IgG (Sigma) under saturating conditions and washed three times in PBS–3% BSA. Pansorbin–Ab complexes were added to viruses complexed with the mouse mAbs, and after incubation at room temperature for 30 min with rocking, virus Ab–Pansorbin complexes were precipitated by centrifugation at 2000 × ***g*** for 30 min. The supernatants were collected and incubated with HSB-2 cells at 37°C for 1 h (virus infection). At 48-h post-incubation, the cells were collected and lysed for immunoblotting. The residual virus content in the supernatant was determined by immunoblotting of HHV-6 immediate early protein.
